# 2163. Prevalence and geographic distribution of mutations in TonB-dependent receptors in the Prospective Observational Pseudomonas aeruginosa (PA) Study

**DOI:** 10.1093/ofid/ofad500.1786

**Published:** 2023-11-27

**Authors:** Stephanie Egge, Rodrigo de Paula Baptista, Cesar A Arias, Vincent Tam, Michael J Satlin, William R Miller

**Affiliations:** Oregon Health Sciences University, Portland, Oregon; Houston Methodist Hospital, Houston, Texas; Houston Methodist and Weill Cornell Medical College, Houston, TX; University of Houston, Houston, Texas; Weill Cornell Medicine, New York, NY; Houston Methodist Research Institute, Houston, Texas

## Abstract

**Background:**

Cefiderocol (FDC) is a last line treatment for carbapenem-resistant Pseudomonas aeruginosa (CR-PA). FDC retains activity against CR-PA by inhibition of penicillin-binding proteins facilitated by active uptake of drug via TonB-dependent receptors (TBDR). Prior, we have shown an association between mutations in TBDR genes and loss of FDC susceptibility, particularly in association with changes at the PA-derived cephalosporinase (PDC/AmpC) active site. Here, we outline geographic variation and prevalence of TBDR mutations in the Prospective Observational Pseudomonas (POP) study.

**Figure 1.**
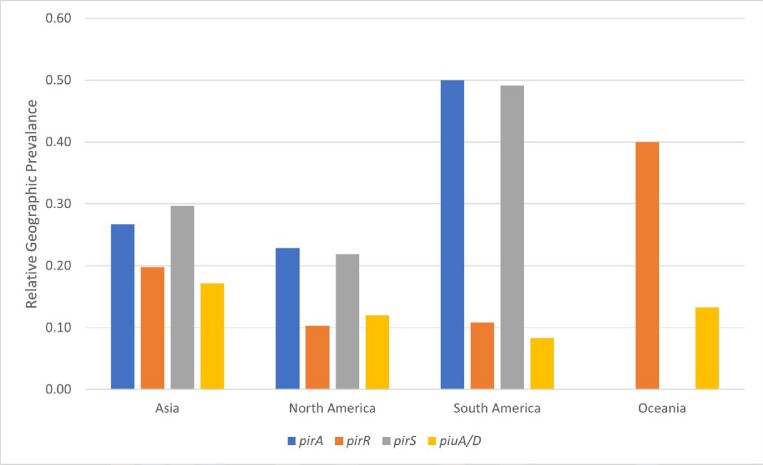
SNP frequency in the pirR, pirS, pirA, and piuA/piuD genes across geographic location.

**Figure 2.**
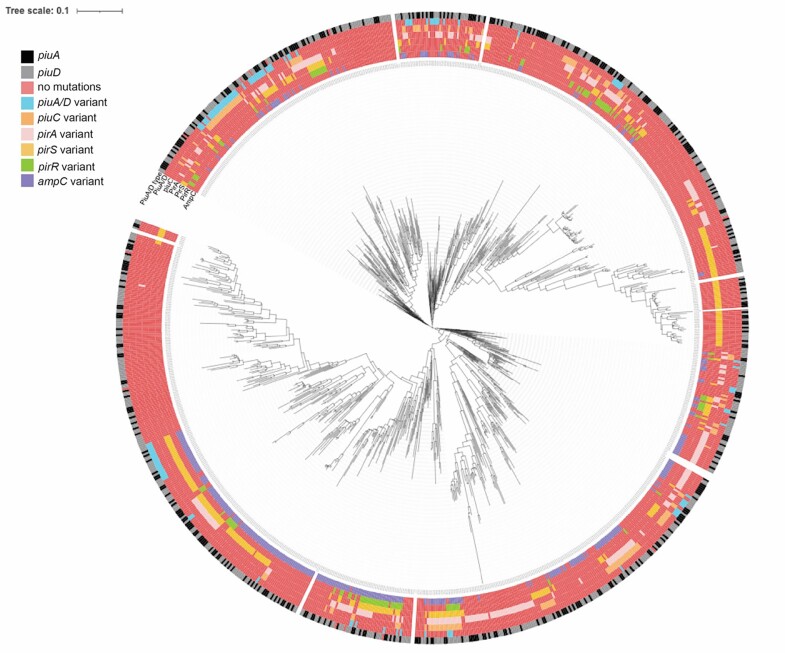
Genotypic variation of PirRS and PiuA/D genes in the 972 P. aeruginosa isolates of the POP cohort.

Black versus gray differentiates presence of either PiuA versus PiuD homologous proteins. Color legend corresponds to presence of TBDR gene variants/SNPs per gene pirR, pirS, pirA, piuA/piuD, and ampC.

**Methods:**

The genomes of 972 CR-PA isolates collected across 10 countries in North America, South America, Asia, and Oceania were screened for single nucleotide polymorphisms (SNP) resulting in missense, insertion/deletion (indel), frameshift, and early stop codon mutations in the TBDR genes *pirR*, *pirS*, *pirA*, *piuA/piuD*, and *ampC* using PAO1 as a reference. SNPs were defined as variant mutations occurring in ≤10% of isolates. A core genome maximum likelihood phylogenetic tree was used to assess distribution and prevalence of genotypic variants. Neighbor-joining trees of the variants in each gene were used to analyze clusters across lineages.

**Results:**

Across all regions except Oceania, SNPs were most frequently seen in *pirS* and *pirA* (**Fig 1**). Gene variants comprised from 8% up to 50% of each geographic population. On phylogenetic mapping, isolates within the same lineage tended to have clustering of shared mutations across the TBDR genes *pirS, pirR*, and *pirA*, and the majority of *piuA*/*piuD* mutations clustered within two distinct lineages (**Fig 2**). Major mutations, including indel, frameshift, and early stop codon mutations occurred in 16 isolates (1.6%), predominantly in isolates from the United States (n=14). Variations in PDC were frequently observed, with 57 different variants across 35.8% of the population. Seven of the PDC variants (n=10 isolates) have been associated with decreased susceptibility to ceftolozane/tazobactam, but these variants did not co-occur with major TBDR mutations.

**Conclusion:**

TBDR mutations occur in PA isolates worldwide. Mutations previously associated with reduced susceptibility to FDC occurred most frequently in isolates from the United States.

**Disclosures:**

**Michael J. Satlin, MD**, AbbVie: IDMC member|Biomerieux: Grant/Research Support|Merck: Grant/Research Support|Shionogi: Advisor/Consultant|SNIPRBiome: Grant/Research Support **William R. Miller, M.D.**, Merck: Grant/Research Support|UpToDate: Honoraria

